# Academic Performance and Resilience in Secondary Education Students

**DOI:** 10.3390/jintelligence13050056

**Published:** 2025-05-16

**Authors:** Ana María Carroza-Pacheco, Benito León-del-Barco, Carolina Bringas Molleda

**Affiliations:** 1 Centro Asociado UNED Mérida, National University of Distance Education, 06800 Mérida, Spain; 2 Department of Psychology and Anthropology, University of Extremadura, 10003 Cáceres, Spain; cbringas@unex.es

**Keywords:** academic performance, resilience, secondary education, students, personal factors

## Abstract

Academic performance is a factor of concern and interest in the educational context for the improvement of the educational and economic system of any country. Determining the factors influencing it has been the subject of multiple investigations. This study focused on analysing which dimensions of school resilience could act as determinants of academic performance in a sample of 609 Spanish secondary education students, aged between 11 and 17 years. The School Resilience Scale (SRS) was used as a data collection instrument. The data were analysed using analysis of variance and discriminant analysis based on a canonical function model, which suggested the existence of a direct and significant relationship between academic performance and all dimensions of resilience, with somewhat larger effect sizes for the Internal Resources and Identity–Self-Esteem dimensions, which allowed us to classify students with particularly high levels of performance. The results also show that the school year was significantly associated with academic performance, with the highest percentages of students at the highest level observed in the 2nd and 3rd years.

## 1. Introduction

Academic performance is the result of students’ school achievements and is influenced by factors such as family, peer group, economic possibilities, teachers, classmates, motivation, and interest in the subject activities (Cano Celestino and Robles Rivera 2018). Other authors define it as the set of students’ skills and abilities to demonstrate their knowledge in different areas (Bestué-Laguna and Escolano-Pérez 2021), or as a measure of what students have learnt during the educational process, evidenced through planned evaluation procedures (Ariza et al. 2018; Niño-Tezén et al. 2024). Academic performance is thus commonly evaluated through exam scores, as part of a broader definition that includes school grades and demonstrated learning (Menéndez-Aller et al. 2021).

In any case, academic performance is a factor of great concern and interest in the educational context, not only for improving a country’s educational and economic systems (Bestué-Laguna and Escolano-Pérez 2021; Frutos de Miguel 2025), but also because of its individual-level implications. Academic performance is closely linked to the development of students’ self-esteem and sense of self-efficacy, as it often represents the first external benchmark through which they are evaluated outside the family environment. Moreover, it is a complex process involving pedagogical and academic variables, teaching strategies, and intrinsic factors. It has proven to be a significant predictor of achievement not only during the educational stage, but also in later stages of life (Niño-Tezén et al. 2024; Wolff et al. 2018).

On the other hand, academic performance has traditionally been associated with students’ prior intellectual capacity. However, empirical evidence has shown that being cognitively intelligent is not enough to guarantee academic success (Fernández-Berrocal et al. 2017). Consequently, recent investigations have explored a wide range of variables related to the educational context and their potential relationships with academic performance. These variables pertain not only to the teaching staff and the broader educational community, but also to students themselves (for example, emotional competencies, relationship skills, critical thinking, motivation, coping, self-efficacy, and self-concept), and they tend to correlate positively with academic performance, while stress and perceived stress typically show negative correlations (Ahmad et al. 2019; Artunduaga 2024; Atkins et al. 2023; Ayala and Manzano 2018; Espinosa-Castro et al. 2020; Guevara-Dávila et al. 2019; Paechter et al. 2022; Ros-Morente et al. 2017; Tipismana 2019). Other contributing factors include family characteristics, such as high parental education, middle or privileged occupational class, and minimal direct help with homework but high academic expectations, which are also associated with higher performance (Fajardo-Bullón et al. 2017). Interpersonal relationships in the classroom, including approval, instrumental support, and affection from teachers, and satisfaction, acceptance, and companionship with peers, also show positive associations (Cuadros and León-del Barco 2024).

Notwithstanding the above, the variable that has received the most attention recently in terms of its relationship with academic performance has been resilience, not only in university students (Bittmann 2021; Gómez-Esquivel et al. 2021; Morgan-Asch 2021; Zumárraga-Espinosa 2023), but also in preadolescents and adolescents (Frutos de Miguel 2025; García-Crespo et al. 2022; Suárez-Cretton and Castro-Méndez 2022; Supervía et al. 2022), although not all the research has confirmed the relationship between resilience and academic performance (Hernández-Muñoz et al. 2024; Niño-Tezén et al. 2024).

Despite this broad and growing body of literature, relatively few studies have examined how resilience (especially in its multidimensional conceptualizations) contributes to explaining academic performance in school-aged populations (e.g., Morris et al. 2021; Obradović et al. 2010). Most existing works focus either on isolated personal factors (e.g., Burton 2020) or on university-level students (e.g., Martin and Marsh 2008), thus leaving a gap in understanding how resilience interacts with a wider set of educational and psychosocial variables in earlier academic stages. This study addresses this gap by exploring resilience as a core explanatory factor within a complex framework that includes both personal and contextual influences.

As for resilience, it is widely defined as a dynamic process that emerges following exposure to stressful, challenging, or adverse experiences, enabling individuals to adapt positively (Frias et al. 2020; Haktanir et al. 2021; Rosales-Pérez 2023). However, conceptualizations vary: some authors describe it as a human skill that allows individuals to face adversity and live productively (Oducado et al. 2021); others describe it as an emotional-intelligence-related ability that helps preserve well-being in the face of threats (Waugh and Sali 2023). Although it has become a topic of interest across disciplines, there is no single definition; what prevails is the view of resilience as a dynamic, multidimensional process oriented towards resource optimization and positive adaptation despite adversity (Kotliarenco 2021).

Two primary conceptual frameworks dominate the current literature on resilience. The first includes developmental models, which examine how individual and socio-contextual factors contribute to patterns of risk and adaptation across the lifespan (Cicchetti and Toth 2009). These models emphasize the interplay between personal attributes and protective contexts. For instance, resilience is associated with internal traits, such as problem-solving abilities, emotional regulation, and empathy, as well as with external supports, like family and significant others (González-Arratia 2016). In educational settings, it is often defined as students’ ability to resist, recover from, and overcome academic setbacks while developing effective coping strategies (Bittmann 2021; Cassidy 2016). Numerous studies show that higher levels of general resilience correlate positively with academic performance (Ayala and Manzano 2018; Bestué-Laguna and Escolano-Pérez 2021; Bittmann 2021; Erdem and Kaya 2021; Supervía et al. 2022). Similarly, research on academic resilience confirms its direct link to better academic outcomes (Abubakar et al. 2021; Agasisti et al. 2021; Dwiastuti et al. 2022; Ononye et al. 2022). Large-scale assessments, like PISA, further support these findings, showing that resilient students often perform above expectations regardless of their socioeconomic background (Dueñas et al. 2019; MECD 2022).

The second major perspective is constructionist, which conceptualizes resilience as a process of seeking and negotiating access to resources across systems, which are themselves more or less resilient (Ungar 2012). This view emphasizes that resilience is not only about individual capacity, but also about the quality and accessibility of surrounding environments. From this standpoint, academic resilience cannot be attributed solely to personal traits; it also depends on the interaction between individuals and their broader context (Tipismana 2019). Family systems (through their beliefs, organization, and communication) play a key role in shaping resilience (Castro et al. 2019). Likewise, school climate has been identified as a crucial protective factor, associated with reduced bullying, better academic performance, and improved psychological well-being (Daily et al. 2019; Farina 2019; Hong et al. 2018; Newland et al. 2019; Varela et al. 2019). Teacher support also contributes significantly to academic resilience (Fang et al. 2020), and a positive school climate can mitigate the impact of socioeconomic disadvantage on academic achievement (Berkowitz et al. 2017; Daily et al. 2020). Furthermore, resilience can be cultivated through targeted programs that promote coping strategies, socio-emotional skills, and the creation of support networks (Mansfield et al. 2021).

These two frameworks, developmental and constructionist, converge on the idea that resilience is shaped by multisystemic protective factors embedded within a person’s social ecology. This integrative, ecological perspective aligns with the approach adopted in the present study.

Taking into account the aforementioned research and following a socio-educational approach, this study aims to analyse which dimensions of school resilience might act as determining factors in the academic performance of secondary education students. This age range is considered a critical period, with a high incidence of school dropout due to academic failure.

## 2. Materials and Methods

### 2.1. Participants

Participants were selected using a multi-stage cluster sampling procedure. First, a number was assigned to each of the 130 secondary education schools in the Extremadura region. Using a computer-generated random number program, three educational centres were randomly selected. In each of the three centres, there were four classrooms per grade, and two classrooms per grade were randomly selected using the same random number procedure.

The sample, with a 95% confidence interval and ±5 margin of error, included 609 secondary education students: 160 (26.3%) in the first year, 140 (23.0%) in the second, 158 (25.9%) in the third, and 151 (24.8%) in the fourth. Of these, 305 (50.1%) were girls and 304 (49.9%) were boys. The participants’ ages ranged from 11 to 17 years, with a mean of 13.43 years (SD = 1.31).

### 2.2. Materials

School Resilience Scale (SRS) (Saavedra and Castro 2009), measures resilience in children aged 9 to 14. It consists of 27 self-administered items on a 5-point Likert scale, with responses ranging from 1 (“Strongly disagree”) to 5 (“Strongly agree”). The total score ranges from 27 to 135 points.

This scale consists, in turn, of five areas or dimensions with a variable number of items, operationally defined as follows:

- The Identity–Self-Esteem Dimension (ISD) refers to internal strengths and more structural aspects of personality (personal identity, self-image, and self-assessment). It is composed of items 1 to 9 (for example, “I am a person who loves myself”).
- The Networks–Models Dimension (NMD) refers to the perception of support, emotional networks, social networks, orientation, and perception of goals. It is composed of items 10 to 18 (for example, “I have a family that supports me”).
- The Learning-Generativity Dimension (LGD) refers to the possibilities of expression, seeking help, facing difficulties, and learning capacity, among others. It is composed of items 19 to 27 (for example, “I can talk about my emotions with others”).
- The Internal Resources Dimension (IRD) refers to the resources and conditions born from the subject in the construction of the response. It is composed of items 1, 2, 3, 5, 7, 8, 9, 16, 17, 18, 20, 26, and 27 (for example, “I am optimistic about the future”).
- The External Resources Dimension (ERD) refers to interactional aspects with the environment that intervene in the construction of resilient behaviour. It is composed of items 4, 6, 10, 11, 12, 13, 14, 15, 19, 21, 22, 23, 24, and 25 (for example, “I feel safe in the environment in which I live”).

For our data, the reliability indices were the Identity–Self-Esteem Dimension (ISD), with Cronbach’s alpha (α = 0.78) and McDonald’s omega (Ω = 0.78); the Networks–Models Dimension (NMD), with. Cronbach’s alpha (α = 0.83) and McDonald’s omega (Ω = 0.84); the Learning-Generativity Dimension (LGD), with Cronbach’s alpha (α = 0.81) and McDonald’s omega (Ω = 0.82); the Internal Resources Dimension (IRD), with Cronbach’s alpha (α = 0.84) and McDonald’s omega (Ω = 0.84); the External Resources Dimension (ERD), with Cronbach’s alpha (α = 0.89) and McDonald’s omega (Ω = 0.89); the total SRS score, with Cronbach’s alpha (α = 0.91) and McDonald’s omega (Ω = 0.91). These indices demonstrate very good internal consistency.

To determine whether the structure found by the authors in the original scale was maintained with our data, we performed a confirmatory analysis, using the goodness-of-fit indices described in Table 1. As can be seen, the fit indices are close to the desirable values, showing evidence of validity for the generalisation of our results.

Academic Performance: This was estimated as an observed variable, calculated as the average of the student’s marks and corresponding to the grades obtained in the last quarter in the four subjects common to all courses (Spanish Language and Literature, Mathematics, English Language, and Geography and History). This variable reports numerical information with ranges from 1.0 to 10.0 with one decimal at the maximum.

### 2.3. Procedure

Data collection was carried out on dates previously agreed upon by the directors and tutors of the centres and in the respective classrooms of each course and group of participants, taking advantage of the tutoring hours that they had marked in their school calendar. The duration of each session was approximately one hour, including the presentation of the test they had to take and the instructions for the proper completion of the booklets, placing special emphasis on the need to answer all the items, and to do so sincerely.

Prior to these interviews, a pilot test was conducted with some secondary education students aged between 11 and 16, in order to estimate the average time required to complete the test properly, as well as to check whether the wording of the items was in accordance with their levels of understanding. The results of this test show that, in all cases, the time required was less than 25 min, and that doubts about the statements were few and easily resolved by the researcher and her team.

Participating students did so voluntarily and without any reward, with the prior informed consent of their parents or legal guardians, as well as the directors of the respective centres.

Despite the explicit instructions given to the participants in each and every session, both at the beginning of the sessions and when they handed in the completed booklets, a certain number of unanswered items were found in some of these. Following the usual standardised procedure, the booklets that had a percentage of unanswered items equal to or greater than 10% were discarded. For participants who had left a smaller number of unanswered items, the mean imputation based on the sample was assigned.

### 2.4. Data Analysis

Initially, reliability analyses (Cronbach’s Alpha and McDonald’s Omega) and confirmatory analysis of the SRS were performed to determine whether the conceptual structure of the SRS, described in the original studies, adequately fit our data.

Subsequently, three statistical analyses were carried out in this study:

(1) ANOVAs with partial eta squared was used to assess effect size. The data were subjected to the Kolmogorov–Smirnov test to analyse the normal distribution, finding *p* > .05 and normality for all observed variables. Likewise, the *p* value > .05 obtained in the Levene test demonstrated the equality of the variances of the groups.

(2) Discriminant analysis was used to analyse which dimensions of the School Resilience Scale discriminated the different levels of academic performance. The assumptions of linearity and normal distribution were taken into account to perform the discriminant analysis. The data were subjected to the Kolmogorov–Smirnov test to analyse the normal distribution, finding *p* > .05 and normality for all the variables observed. Likewise, the value *p* > .05 obtained in Box’s M test demonstrated the equality of the covariance matrices of the groups.

In this analysis, academic performance was included as a dependent variable, grouped into three levels: low (>6), medium (≥6 and <8), and high (≥8). The dimensions of the SRS were considered as independent and predictive variables: (1) Identity–Self-Esteem Dimension (ISD); (2) Networks–Models Dimension (NMD); (3) Learning-Generativity Dimension (LGD); (4) Internal Resources Dimension (IRD); and (5) External Resources Dimension (ERD).

(3) To complete the discriminant analysis and to determine the role that the participants’ school year might play, a classification model based on flow diagrams was created using the decision tree statistical technique.

Statistical analyses were performed with SPSS statistical package version 21.0 for PC and Free JASP.

## 3. Results

Initially, the possible existence of differences among the means of the three levels of academic performance in terms of the scores of the dimensions in the School Resilience Scale was examined. For this purpose, analysis of variance (ANOVA) was performed, finding significant differences among the three levels of performance for the following dimensions (Table 2): Identity–Self-Esteem (ISD; F = 23.060, *p* = .000); Networks–Models, (NMD; F = 12.772, *p* = .000); Learning-Generativity (LGD) (F = 12.692, *p* = .000); Internal Resources (IRD; F = 25.578, *p* = .000); and External Resources (ERD; F = 10.375, *p* = .000). Values of the effect size, the partial eta squared, indicate a mild–moderate effect.

Once the existence of differences among the means of the three groups of academic performance levels was established, a discriminant analysis was performed to identify which dimensions of the SRS best explain these differences. Table 3 presents the structure matrix resulting from this analysis. Among the three discriminant functions calculated, Function 1 emerged as the most significant in distinguishing between the performance levels.

Function 1 accounted for the highest proportion of variance among the three functions, exhibited the strongest canonical correlation, and yielded the smallest Wilks’ Lambda value, indicating a greater degree of separation among the groups. The chi-squared test for this function also reached the highest level of statistical significance (Function 1: % of variance = 86, canonical correlation = 0.280, Wilks’ λ = 0.909, χ^2^ = 57.752, df = 8, *p* < .001).

This means that Function 1 plays a key role in differentiating students based on their academic performance, primarily through two variables: Internal Resources (IRD) and Identity–Self-Esteem (ISD). In practical terms, students with higher academic performance tend to score higher on these two dimensions, which suggests a stronger internal capacity to cope with academic demands and a more consolidated personal identity.

In Table 4, it can be observed that the canonical discriminant function correctly classified 40.2% of students with a low academic performance level, 31.2% with a medium level, and 58.91% with a high level. The average gains in prediction were higher than the 33% accuracy expected at random across the three performance levels. These percentages indicate that these dimensions of the SRS help us, mainly, to discriminate among high-level students.

Finally, with the intention of analysing the role of the school year in the associations found, a classification tree was created, considering as a dependent variable the low and high levels of academic performance. As seen in the classification results of the discriminant analysis, the associations of the dimensions of the SRS used as predictors were useful in the group with a low level of performance and especially in the high level. The following independent variables were introduced: dimensions of the SRS and the school year.

**Figure 1:**
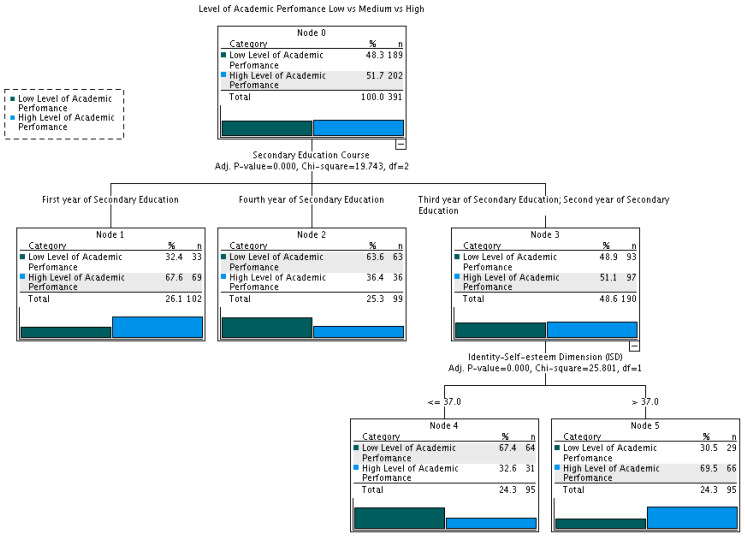
Decision tree for the dependent variables: low level and high level of academic performance.

As can be seen in Figure 1, the school year was significantly associated with academic performance. The highest percentage of students in the high level of academic performance (69.5%) was found in students in their 2nd and 3rd years of secondary education and students who obtained higher scores in Identity–Self-Esteem (ISD; Node 5).

## 4. Discussion

The main objective of the present study was to analyse which dimensions of school resilience could act as determining factors in the academic performance of secondary education students. Based on the results obtained, there was a direct and significant correlation between academic performance and all dimensions of the School Resilience Scale (SRS; (Saavedra and Castro 2009)), with slightly larger effect sizes found for Internal Resources (IRD) and Identity–Self-Esteem (ISD). This pattern is consistent with the findings of Suárez-Cretton and Castro-Méndez (2022), who, using the same instrument (SRS) with Chilean students aged 9–14, also found stronger associations between these particular dimensions and academic achievement. Their methodology involved intentional sampling of entire classroom groups in public schools with high levels of vulnerability, applying the SRS and using first-semester grades in Language and Mathematics as the performance indicators.

Our findings also align with previous research confirming the positive relationship between academic performance and general or academic resilience in adolescents (Frutos de Miguel 2025; García-Crespo et al. 2022; Supervía et al. 2022), and even in university populations (Dwiastuti et al. 2022; Ononye et al. 2022; Zumárraga-Espinosa 2023). For instance, Erdem and Kaya (2021) employed the Academic Resilience Scale and found significant associations between resilience and academic achievement among Turkish high school students, using a sample of 390 students and analysing their average GPA. Similarly, Frutos de Miguel (2025) investigated a Spanish sample of early adolescents and confirmed the predictive role of resilience when measured alongside motivational and self-concept variables. These studies used different methodological approaches but converged in identifying resilience as a relevant factor in educational outcomes.

Moreover, the results of Supervía et al. (2022) highlighted the role of academic self-efficacy as a mediator between resilience and academic performance. Their study, which used a sample of Spanish adolescents and a combination of validated resilience and self-efficacy instruments, found that while the direct relationship between resilience and academic performance was modest, it became significant when mediated by self-efficacy. This supports an ecological interpretation of resilience, whereby internal psychological resources serve as mechanisms linking broader resilient traits with academic outcomes.

The results of the present study further show that resilience explained 58.9% of the variance in high academic performance levels among secondary education students. This supports the notion of resilience as a predictive indicator, as suggested in previous research (Ayala and Manzano 2018; Bestué-Laguna and Escolano-Pérez 2021).

However, these findings are in contrast to other studies that did not find such a correlation. For example, Hernández-Muñoz et al. (2024), working with general basic education students, reported only a slight relationship between general resilience and academic performance. Similarly, Alonso-Aldana et al. (2016) and Niño-Tezén et al. (2024) found no significant relationships in university samples. These discrepancies could stem from uncontrolled mediating or moderating variables, such as academic satisfaction, self-efficacy, self-concept and motivation, peer relationships, or emotional resilience (Dwiastuti et al. 2022; Etherton et al. 2020; Frutos de Miguel 2025; Liew et al. 2018; Ononye et al. 2022).

Supporting this, the review conducted by Redondo-Blasco and Martínez-Abad (2024) synthesised 22 studies published after 2017 on the resilience–academic performance relationship in compulsory education. Their findings confirmed a generally positive, significant, and direct correlation, while also emphasising the influence of various mediating, moderating, and contextual factors.

Our discriminant analysis further identified the most predictive dimensions of resilience in terms of academic performance. Internal resources and self-esteem emerged as critical components. Among these, internal resources, such as self-efficacy, self-concept, and motivation, stand out as key protective factors that help students cope with academic challenges more effectively.

Self-efficacy supports optimistic expectations and goal-setting (Paredes-Valverde et al. 2020; Sadoughi 2018), while a positive self-concept enhances confidence in one’s academic capabilities (Haktanir et al. 2021; Wolff et al. 2018). Motivation plays a central role in student engagement and persistence (Núñez et al. 2024; Paechter et al. 2022). These findings align with prior evidence on the importance of internal strengths for academic success (Borja-Naranjo et al. 2021; Martín-Romero and Sánchez-López 2021; Menéndez-Aller et al. 2021; Nájera et al. 2020; Vega-Díaz 2024).

The predominance of internal dimensions, such as IRD and ISD, in predicting academic performance underlines the pivotal role of personal strengths in overcoming academic demands. These findings suggest that educational interventions aimed at boosting internal psychological resources (such as training in self-efficacy, emotional regulation, and self-awareness) could be particularly impactful. Incorporating these aspects into school curricula may not only foster academic success but also long-term personal development.

In terms of self-esteem, it relates to emotional regulation, which facilitates stress management and academic focus (Ros-Morente et al. 2017). Compared to external resources, such as family dynamics (Fajardo-Bullón et al. 2017) or peer interactions (Cuadros and León-del Barco 2024), internal resources demonstrate greater predictive power because they are inherent to the student and directly influence academic behaviour and autonomy.

Our results also indicate that the academic year is significantly associated with performance, with students in their 2nd and 3rd years of secondary education more likely to exhibit high academic performance and high scores in the Identity–Self-Esteem Dimension of the SRS. The higher repetition rate in the first year (7.3%; MECD 2024) may reflect the challenging transition from primary to secondary education (Ávila et al. 2022). Conversely, the lower performance of 4th-year students may relate to a decline in general self-concept with age (Núñez et al. 2024), a phenomenon also linked to academic success (Pérez-Mármol et al. 2023).

### Limitations

As with any study, some limitations of this work must be pointed out. The most important is the use of self-reports as a method of data collection. Self-reporting for the assessment of resilience is a measure subject to the subjective and temporary perception of the students. Other limitations refer to the cross-sectional design, which makes it difficult to establish greater inferences about the relationship between the variables of the study. Finally, it would be optimal to be able to replicate the study by increasing the sample size and including representation at the national and international levels.

Although the students were sampled by clusters (i.e., classrooms), we did not apply multilevel modelling techniques because no classroom-level variables were collected, and all measures were based on student self-reports. A runs test indicated that the assumption of independence was not violated (*p* = .10). However, we acknowledge the potential for unobserved classroom-level effects and recommend that future research implement multilevel approaches when appropriate to better account for hierarchical structures and avoid potential overestimation of effects.

Additionally, the cultural context in which this study was conducted (Spanish secondary education) should be considered when interpreting the findings. Educational practices, resilience constructs, and academic expectations can vary considerably across countries and school systems. As such, the generalizability of these results to other populations may be limited, and replication in different cultural or educational settings is encouraged to validate the external applicability of the conclusions.

## 5. Conclusions

This study examined the relationship between academic performance and school resilience in secondary education students. Differences were found among the means of the three performance levels based on scores across the five resilience dimensions. Discriminant analysis revealed a canonical function in which the most predictive dimensions of high performance were Internal Resources and Identity–Self-Esteem. This approach has provided a clear view of the variables that contribute to resilience and may help guide the design of more effective interventions to improve students’ academic performance. Furthermore, analysis of the role of the school year in the identified associations showed that this factor was significantly related to academic performance, with the highest percentages of students at the highest level observed in the 2nd and 3rd years. Taken together, these findings, along with those from the recent research on academic performance cited above, highlight the relative importance of certain personality variables on students’ achievement levels. There is also a growing interest in promoting resilience among young people.

## Figures and Tables

**Table 1:** Goodness-of-fit indices of the proposed model, School Resilience Scale (SRS).

Model	χ²	χ²/df	GFI	IFI	TLI	CFI	RMSR	RMSEA
3 related factors and 2 second-order factors	1058.976	3.664	0.989	0.900	0.864	0.888	0.056	0.060

Notes: χ² = chi-squared statistic; χ²/df = chi square divided by degrees of freedom; GFI = goodness-of-fit index; IFI = incremental fit index; TLI = Tucker–Lewis index; CFI = comparative goodness-of-fit index; RMSR = root mean square residual; RMSEA = root mean square residual of approximation.

**Table 2:** ANOVA test results: means and standard deviations of the dimensions of the SRS based on different levels of academic performance.

	Academic Performance Levels				ANOVA Test	
SRS Dimensions	Low Level *M (SD)*	Medium Level *M (SD)*	High Level *M (SD)*	*F*	*p*	η^2^ *
ISD	34.91 (5.91)	35.67 (5.52)	38.28 (3.94)	23.060	.000	0.07
NMD	38.01 (5.71)	38.98 (5.85)	40.67 (4.11)	12.772	.000	0.04
LGD	37.83 (5.59)	38.03 (5.41)	40.12 (4.01)	12.692	.000	0.04
IRD	51.66 (8.22)	53.27 (7.33)	56.57 (4.97)	25.578	.000	0.08
ERD	59.08 (8.98)	59.42 (9.06)	62.50 (6.55)	10.375	.000	0.03

**SRS Dimensions:** Identity–Self-Esteem (ISD), Networks–Models (NMD), Learning-Generativity (LGD), Internal Resources (IRD), and External Resources (ERD). * Effect size test, partial eta squared: if 0.06 ≤ η^2^ < 0.14, the effect is moderate; if η^2^ ≥ 0.14, the effect is strong.

**Table 3:** Structure matrix. The variables are ordered by the size of the correlation with the discriminant function.

Variables	Functions	
	Function 1	Function 2
Internal Resources (IRD)	0.997 *	−0.079
Identity–Self-esteem (ISD)	0.944 *	0.184
Networks–Models (NMD)	0.703 *	−0.134
Learning-Generativity (LGD)	0.686 *	0.369
External Resources (ERD)	0.622 *	0.321

* Highest absolute correlation between each variable and the discriminant function.

**Table 4:** Classification results using the discriminant function.

		Predicted Membership Group		
	Performance Levels	Low	Medium	High
%	Low	40.2	21.2	38.6
	Medium	29.4	31.2	39.4
	High	20.8	20.3	58.9

## Data Availability

The datasets generated and/or analysed during the current study are not publicly available due to ethical and legal restrictions related to the confidentiality of participant information. Access to the data is therefore limited in order to protect the privacy of the individuals involved.
